# The Roles of CD73 in Cancer

**DOI:** 10.1155/2014/460654

**Published:** 2014-07-14

**Authors:** Zhao-wei Gao, Ke Dong, Hui-zhong Zhang

**Affiliations:** Department of Clinical Diagnosis, Tangdu Hospital, Fourth Military Medical University, Xinsi Road, Xi'an, Shanxi 710038, China

## Abstract

Purinergic signaling has emerged as an important player in cancer progression and is regulated by a series of nucleotidases. Among the enzyme cascade, CD73, which catelyzes AMP breakdown to adenosine, has been found to be overexpressed in many types of cancer. Various factors and mechanisms are employed to regulate expression of CD73. Accumulating studies have shown that CD73 is a key regulatory molecule of cancer cells proliferation, migration and invasion *in vitro*, tumor angiogenesis, and tumor immune escape *in vivo*. With such important roles in cancer, CD73 has become an appealing therapy target. Recent evidences in mice models demonstrated that targeted blockade of CD73 could be a favorable therapeutic approach for cancer patients in the future. In this review, we will summarize the multiple roles of CD73 in cancer development, including its clinical significance, its promotive effects on tumor growth, metastasis, and angiogenesis, and its suppressive effects on immune response, regulatory mechanisms of CD73 expression, and current situation of anti-CD73 cancer therapy.

## 1. Introduction

Tumorigenesis, progression, and metastasis are complex malignance process* in vivo* that involves a series of events, including rapid proliferation of mutant cells in an uncontrollable manner, inhibition of programmed cell death, abundant angiogenesis, escaped from immune surveillance, invasion and colonization into distant organs. Many signaling pathways have been identified to be involved in cancer progression. Recent years, the purinergic signaling pathway, where the extracellular ATP (adenosine triphosphate), ADP (adenosine diphosphate), and adenosine act as the main signaling molecules, has emerged as an important player in cancer progression [1]. Purinergic signaling is a multistep coordinated cascade, including stimulated release of ATP/ADP, triggering of signaling events via P2 receptors, and then nucleotide inactivated to adenosine. Further, adenosine binds to its own activated P1 receptors and influences biological pathways, such as cell survival, proliferation, and cell motility [2]. More importantly, it is now evident that the adenosine is one of the most important immunosuppressive regulatory molecules in the tumor microenvironment [3].

Nucleotides are released by a variety of cell types in response to multiple stress signals, such as injury, hypoxia and inflammatory condition, etc. Further, the nucleotides are hydrolyzed by the enzyme cascade as follows: ATP/ADP into AMP by NTPDases, AMP into adenosine by ecto-5′-nucleotidase (known as CD73), and adenosine into inosine by adenosine deaminase [4]. Therefore, purinergic signaling is regulated by a series of cell surface-located ectonucleotidases. The balance between ATP/ADP, AMP, and adenosine is crucial in the control of tumor progression.

CD73 is a 70-kD, glycosylphosphatidylinositol (GPI) anchored cell surface protein that is encoded by NT5E gene, also known as ecto-5′-nucleotidase (ecto-5′-NT, EC 3.1.3.5), plays a crucial role in switch on adenosinergic signaling. CD73 has both enzymatic and nonenzymatic functions in cells [5]. As a nucleotidase, CD73 catalyzes the hydrolysis of AMP into adenosine and phosphate. Notably, CD73-generated adenosine plays an important role in tumor immunoescape [6]. In addition to its enzymatic function, CD73 is also a signal and adhesive molecule that can regulate cell interaction with extracellular matrix (ECM) components, such as laminin and fibronectin, to mediate cancer invasive and metastatic properties [7]. Indeed, the enzymatic and nonenzymatic functions of CD73 are both involved in cancer associated process and not completely independent of each other.

CD73 has been found to be overexpressed in many types of cancer cell lines and patient's biopsies including breast cancer, colorectal cancer, ovarian cancer, gastric cancer, and gallbladder cancer and associated with clinical characteristics, or prognosis of cancer patients (Tables 1 and 2). Increasing evidence has verified that CD73 is a key regulatory molecule in cancer development [27]. In particular, due to the favorable effect on tumor-bearing mice models, although have not been investigated in clinical patients, anti-CD73 therapy has become a promising approach for the treatment of cancer patients in the future [28, 29]. In this present paper, we will summarize the roles of CD73 in cancer development, including its clinical significance in cancer patients, its promotive effects on tumor growth, metastasis, and angiogenesis, and its suppressive effects on immune system under tumor microenvironment, regulation mechanisms of CD73 expression, and opportunities for anti-CD73 cancer therapy in the future.

## 2. Clinical Significance of CD73 in Cancer Patients

Overexpression of CD73 has been observed in broad types of cancers, and its clinical significance has also been found by correlative analysis (Table 2). In breast cancer, Loi et al. demonstrated that CD73 expression was significantly associated with a worse prognosis in triple negative breast cancer (TNBC) patients (*n* = 661, *P* = 0.029) but not in patients with luminal (*n* = 2083, *P* = 0.7) or HER2^+^ (*n* = 487, *P* = 0.86) breast cancer [26]. In contrast, another retrospective study (*n* = 136) reported that positive CD73 expression was strongly correlated with longer disease-free survival (*P* = 0.0044) and overall survival (*P* = 0.027) of breast cancer patients [25], which suggested that elevated CD73 expression could predict a good prognosis in stages I–III breast cancer patients. Notably, the prognostic implication of CD73 expression in breast cancer remains controversial and is not independent of other clinical indexes. Now, a retrospective analysis by our team in more clinical specimens is in process, which will take more factors into consideration, such as patient's age, subtype, and population, treatment.

In digestive system cancers, recently, Lu et al. evaluated the clinical significance and prognostic value of CD73 in human gastric cancer (*n* = 68 patients): analysis of CD73 expression by immune histochemistry (IHC) revealed that overexpression of CD73 was positively correlated with differentiation of tumor, depth of invasion (*P* < 0.001), nodal status (*P* = 0.003), metastasis (*P* = 0.013), and the cancer stage (*P* < 0.001), and the overall survival rate was low in the patients with high expression of CD73 (*P* < 0.001) [19]. In addition to gastric cancer, other studies showed that high levels of CD73 in colorectal cancer (CRC) patients were correlated with a poor prognosis [16, 17]. Moreover, Xiong et al. recently reported that CD73 expression was associated with tumor progression and survival of patients with gallbladder cancer [20].

In hematologic neoplasm, Zhao et al. investigated the expression of CD73 in various leukemia subtypes (*n* = 86) and revealed that the expression of CD73 was associated with leukemia subtype, differentiation, and development [21]. Serra et al. assessed the clinical implication of CD73 in chronic lymphoblastic leukemia (CLL, *n* = 299) and found that high expression of CD73 was associated with a more aggressive clinical behavior [22], whereas another study showed that CD73 expression had no prognostic value in children (1–18 years old) with acute lymphoblastic leukemia (ALL, *n* = 338) [23].

In other cancers, Yang et al. reported that overexpression of CD73 in prostate cancer was associated with lymph node metastasis [24]. And Oh et al. showed that overexpression of CD73 in epithelial ovarian carcinoma was associated with better prognosis, lower stage, and better differentiation [18]. Wang et al. found that CD73 expression in malignant melanoma was associated with metastatic site specificity [9]. Taken together, these results supported that CD73 was an important clinical or prognostic biomarker in several types of cancer, which presented the potential value of CD73 for clinical diagnosis and prognosis.

## 3. CD73 and Drug Resistance

In addition to being as a clinical and prognostic marker in cancer patients, overexpression of CD73 has also been found to be associated with resistance to antitumor agents. Actually, as far as in 1996, Ujhazy et al. firstly reported that the chemoresistant phenotype of cell lines (MDR positive) was correlated with a coexpression of elevated CD73 levels [30]. Recently, Quezada and his colleagues reported that inhibition of CD73 activity or knocking down CD73 expression by siRNA reversed the vincristine resistance phenotype of glioblastoma multiforme (GBM) cells [8]. Notably, in GBM tissues and cells, CD73 was cooverexpressed with multiple drug protein-1 (Mrp1) which was the most important transporter conferring multiple drug resistance in tumor cells [8, 31]. Consistently, knockdown of CD73 expression by siRNA in GBM cells reduced Mrp1 expression [8]. These results indicated that overexpression of CD73 might mediate vincristine resistance in GBM cells through regulating the expression of Mrp1.

Recently, Loi et al. evaluated the connection between CD73 expression level and pathologic complete responses (pCR) rate in triple negative breast cancer patients (*n* = 59) treated with anthracycline-only preoperative chemotherapy. Their result showed that low CD73 expression was significantly associated with an increased pCR rate (*P* = 0.00002) [26]. Furthermore, by using mouse models of breast cancer, they demonstrated that CD73 overexpression in tumor cells conferred chemoresistance to doxorubicin dependent on the activation of A2A adenosine receptor. Not surprisingly, targeted blockade of A2A adenosine receptors could rescue doxorubicin sensitivity of CD73-overexpressing tumors in mice models.

Moreover, despite the growing interest of cancer immunotherapy, clinical benefits are still modest because the mechanisms of immune escape. As a key regulatory molecule of immune escape* in vivo*, CD73 is certainly involved in immunotherapy resistance [12]. Thus, as the link between CD73 and resistance to some antitumor therapy, combining anti-CD73 treatment with chemotherapy or immunotherapy may be an effective approach for these patients with high CD73 levels. In the future, while in the coming age of individual cancer therapy, CD73 expression in cancer patients may be served as a detectable gene marker to choose and use applicable drugs for cancer treatment.

## 4. CD73 and Tumor Growth 

Overexpression of CD73 has been found to promote cancer cells proliferation* in vitro*. Zhi et al. observed that expression suppression of CD73 by shRNA could inhibit proliferation of breast cancer cells (MB-MDA-231) via inducing cell-cycle arrest and cell apoptosis [32]. In addition, treatment with APCP (*α*, *β*-methylene adenosine-5′-disphosphate), which is a specific inhibitor of CD73 enzymatic activity, can also inhibit cancer cells proliferation in a dose-dependent manner [32–34]. Correspondingly, in pcDNA-NT5E transfected breast cancer cells (MCF-7), CD73 overexpression increased cell viability and promoted cell-cycle progression. Bavaresco et al. found that APCP treatment caused a significant reduction of 30% in glioma cell proliferation while treatment with adenosine increased cell proliferation by 35%. Together, these results supported that CD73 promote cancer cells growth, dependent on its enzymatic activity, that is, production of adenosine [35].

However, there were conflicting studies which showed that adenosine can also inhibit cell growth and induce apoptosis. Wang and Ren found that extracellular adenosine induce apoptosis of gastric carcinoma cells in a dose-dependent manner, and this effect can be decreased by the inhibition of adenosine uptake using the nucleoside transport blocker—dipyridamole [36]. Recently, Shirali et al. found that adenosine could induce apoptosis of ovarian cancer cells via Bax up-regulation and caspase-3 activation which are both crucial apoptotic regulatory molecules [37]. These results suggested that adenosine induce apoptosis dependent on the uptake of adenosine into cells and activation of intracellular apoptotic pathways. Thus, the promotive effect of CD73 on cancer cells proliferation* in vitro* may via other molecule which independent with adenosine, such as EGFR, which is a key molecule involved in cell growth. Recently, Zhi et al. have found the regulative effect of CD73 on EGFR expression and phosphorylation in human breast cancer [38].

*In vivo*, overexpression of CD73 in tumor cells can promote subcutaneous tumor growth in mice model. Zhi et al. showed that subcutaneous tumor in nude mice (produced by injecting with pcDNA-NT5E transfected MCF-7 cells) grew in a more rapid fashion when compared with parental MCF-7 cells produced subcutaneous tumor. And consistently, CD73 downregulation by siRNA could retard tumorigenicity in murine xenograft models [7, 32]. Notably, besides the CD73 in tumor cells, host CD73 also play important role in cancer development* in vivo*. Stagg et al. reported that CD73 deficiency in mice inhibited the development of 3-methylcholanthrene- (MCA-) induced fibrosarcomas compared with wild-type mice. Similarly, CD73 deficiency also suppressed tumorigenesis in transgenic adenocarcinoma of mouse prostate (TRAMP) mice models [39]. Yegutkin et al. investigated the contribution of host CD73 to tumor growth in melanoma models and found that primary tumors were significantly attenuated in CD73 lacking mice [40]. Taken together, since both host and tumor CD73 contribute to primary tumor growth* in vivo*, the optimal antitumor effect of CD73 blockage drug may require targeting both host and tumor CD73.

## 5. CD73 and Tumor Metastasis

Metastasis is a vicious characteristic of malignance cancers and is the dominating cause of mortality in cancer patients. Therefore, identification of metastasis-promoting molecule is primary importance for anticancer research and development of target drugs. To date, accumulating data have shown that CD73 is associated with tumor metastasis in experimental models and clinical patients.

Acquirement of “invasive phenotype,” which means the lost of cell-cell junction and enhanced cell migration ability in tumor cells, is the prerequisite for cancer metastasis. In pcDNA-NT5E transfected breast cancer T-47D cells, upregulation of CD73 promoted cell migration and invasion, and in the meantime, increased cell mobility by CD73 overexpression could be inhibited by APCP [7, 13, 41]. Notably, mRNA and protein expression levels of EGFR were increased significantly in pcDNA-NT5E transfected T-47D cells [41]. More interestingly, downregulation of EGFR by siRNA resulted in inhibition of migration and invasion activities in pcDNA-NT5E transfected T-47D cells. In another study, researchers found that CD73 expression correlated positively to EGFR expression in MB-MDA-231 cells, and EGFR expression could be decreased by suppressing CD73 expression with siRNA [38]. These observations suggested that CD73 promoted tumor cell migration and invasion via regulating EGFR expression.

Recently, Xiong et al. identified CD73 as a key regulator of epithelial-mesenchymal transition (EMT) in gallbladder cancer, which indicated that CD73 might promote cell migration and invasion via inducing EMT of tumor cells [20]. Interestingly, the dual functions of CD73 seemed to fulfill distinct roles in the cell interaction and mobility on ECM. Sadej and Skladanowski observed that the enzymatic function was primarily involved in invasion of cancer cell, whereas the nonenzymatic action of CD73 was proved to promote cell migration on ECM through activation of focal adhesion kinase (FAK) in melanoma cells [10].

*In vivo*, Stagg et al. showed that CD73-deficient mice were resistant to development of lung metastasis after intravenous injection of B16F10 melanoma cells; moreover, host CD73 deficiency increased tumor antigen-specific T-cell homing to tumors [42]. Similarly, Yegutkin et al. also reported that formation of melanoma metastasis was significantly retarded in CD73-deficient mice [40]. In clinical cancer patients, several retrospective studies revealed that CD73 overexpression in tumor was associated with metastasis of gastric cancer, prostate cancer, and malignant melanoma, respectively [9, 19, 24]. Taken together, above results demonstrated that both host and tumor CD73 significantly contribute to tumor metastasis; importantly, the resistant effect of host CD73 deficiency on tumors metastasis is associated with increased endogenous antitumor immunity.

## 6. CD73 and Tumor Angiogenesis

The uncontrolled proliferation of cancer cells needs abundance of nutrition and oxygen. High density of angiogenesis can support the sustenance for tumor cells growth. In addition to sustenance supply, angiogenesis is also the important pathway for distant invasion of tumor cells. Via infiltrating into immature vessels, tumor cells can be carried into distant organs by blood. Thus, abundant tumor angiogenesis is necessary for cancer growth and metastasis.

Formation of capillary-like tubes by endothelial cells is the basic process during angiogenesis. Wang et al. found that capillary-like structures were formed more in CD73 (+/+) pulmonary microvascular endothelial cells (PMECs) than in CD73 (−/−) PMECs* in vitro*. In particular, the difference was more pronounced when PMECs were cultured in cancer-conditioned medium. Consistent with these results* in vitro*, they also observed that the extent and density of tumor angiogenesis were greater in CD73 (+/+) mice than in CD73 (−/−) mice* in vivo *[43]. These results supported that CD73 contributed to endothelial cells forming new vessels especially in cancer condition.

Recently, Koszalka et al. reported that inhibition of CD73 resulted in impaired angiogenesis and reduced melanoma growth in mice models [44]. Allard et al. investigated the importance of CD73 expression during tumor angiogenesis and the effect of anti-CD73 mAb therapy on angiogenesis. By detecting the formation of capillary-like tubes by human umbilical vein endothelial cells (HUVEC)* in vitro*, they found that CD73 gene silencing by shRNA in HUVEC cells was associated with a significant decrease in tube formation. By using mice models of breast cancer, they found that treatment of tumor bearing mice with either anti-CD73 mAb or APCP decreased tumor angiogenesis [45]. Furthermore, Allard's study found that both tumor and host-derived CD73 were involved in tumor angiogenesis. Tumor-derived CD73 increase VEGF production by tumor cells, while host-derived CD73 is required for optimal angiogenic responses to VEGF. Tumor grown in CD73-deficient mice appeared to have less angiogenesis. These results support that tumor and host CD73 synergistically contribute to angiogenesis under tumor condition* in vivo*. In contrast to above results, another recent study in a model of hind limb ischemia showed that CD73 deficiency had no effect on angiogenesis [46]. Thus, the effect of CD73 on angiogenesis might be different in tumor and nontumor microenvironment.

As the promotive effect of angiogenesis in tumor growth and metastasis, several inhibitors antiangiogenic agent have received FDA approval and been used in clinical, including the bevacizumab (i.e., anti-VEGF mAb). However, patients that initially respond to antiangiogenic treatment ultimately become resistant [47]. The mechanisms may rely on the abnormal activation of VEGF pathway. As mentioned above, CD73 can promote angiogenesis via up-regulation of VEGF expression, which indicated that CD73 contribute to acquired-resistance of anti-VEGF therapy. Indeed, we have detected the elevated CD73 level in a bevacizumab resistant patient. Thus, CD73 may represent new target for treatment to overcome bevacizumab resistance in the future.

## 7. CD73 and Immune Suppression

Immunoescape has been considered as one of the “hallmarks” of cancer. During tumor growth, progression, and metastasis, tumor cells employ multiple pathways to evade immune surveillance [48]. One such mechanism relies on the adenosine signaling [49, 50]. Among purinergic signaling cascade, CD73 catalyzed the hydrolysis of AMP into adenosine; thus it is a key molecule in tumor immunoescape [51]. To mediate its immunosuppressive function, CD73 generated adenosine can bind to four distinct G-protein-coupled receptors: A1, A2A, A2B, and A3. Adenosine can exert effect on immune system through multiple pathways [52, 53].

High concentrations of adenosine could blunt the capacity of nature killer (NK) cells to produce tumor necrosis factor-*α* (TNF-*α*) and interferon-*γ* (IFN-*γ*) and also inhibit the lytic activity of NK cells in an A2A receptor dependent manner, thus limiting the ability of NK cells to mediate the lysis of tumor cells [54]. In addition, macrophages were also affected by adenosine. By binding to A2A receptor expressed on macrophage, adenosine led to release of IL-4 and IL-10 which contributed to suppression of antitumor immune response and promoted tumor growth [52].

Dendritic cells (DCs) are critical component of immune system and bridge innate immunity and adaptive immunity. High concentrations of adenosine are in contract with DCs and skews DCs differentiation towards a distinct cell population (i.e., adenosine-differentiated DCs) via binding to A2B receptor on DCs. Novitskiy et al. reported that adenosine-differentiated DCs expressed high levels of angiogenic, immune suppressor, and tolerogenic factors (VEGF, IL-8, IL-10, COX-2, TGF*β*, and IDO) [55]. Moreover, via binding to A2A receptor on DCs, adenosine strongly inhibited production of IL-12 which was an important antitumor cytokine.

T cells comprise the major constituent of adaptive immunity and help to fight against infections and tumors. Adenosine can impact T cells activity by increasing of intracellular cAMP levels [56]. In addition, adenosine mediated inhibition of IL-2 production in tumor microenvironment prevents clonal expansion of immunologically active antitumor T cells. Jin et al. reported that overexpression of CD73 on tumor cells promoted T-cell apoptosis* in vitro* and inhibited antitumor effect of T-cell* in vivo*, while these impairments could be reversed via knockdown of CD73 expression [12]. Moreover, Ryzhov et al. found that CD73 in tumor was involved in infiltration and accumulation of myeloid-derived suppressor cells (MDSCs) that had been identified as a population of immature myeloid cells with the ability to suppress T-cell activation [57].

Taken together, these results demonstrated that CD73-generated adenosine played a key role in the regulation of inflammatory reactions and immune responses through various pathways. Inhibition CD73 or adenosinergic pathway can be a potential target for cancer immunotherapy in the future.

## 8. Regulation of CD73 Expression in Cancer

As mentioned above, CD73 plays important roles in malignant tumor progression and has been found overexpressed in many types of cancers. How is the expression level of CD73 regulated in tumors? There are several mechanisms can explain this question. Firstly, CD73 expression has been found to be negatively regulated by estrogen receptor (ER) in breast cancer, whereby loss of ER significantly enhances CD73 expression. Thus, CD73 is highly expressed in ER negative than ER positive breast cancer patients and might constitute a promising target for clinical treatment of ER negative breast cancer patients [26]. In contrast to ER, thyroid hormones have been identified as a molecule that can increase CD73 expression in several types of cells (glioma cells, vascular smooth muscle cells, and ventricular myocytes) that belong to nervous and cardiovascular system [58–60].

Secondly, hypoxic microenvironment can drive CD73 expression, since there is a HIF-1*α* (hypoxia-inducible factor-1*α*) binding site (namely, hypoxia response element, HRE) in the CD73 gene promoter, expression level of CD73 is increased under hypoxia condition. Correspondingly, suppression of HIF-1*α* by antisense oligonucleotides or point mutations in the HRE of CD73 gene has been found to inhibit hypoxia-induced CD73 expression [61, 62].

The third, inflammatory factors can also regulate CD73 expression, Brisevac et al. have estimated the effect of several inflammatory factors (LPS: lipopolysaccharide, TNF-*α*: tumor necrosis factor-alpha, IFN-*γ*: interferon-gamma, and Glu: glutamate) on CD73 expression. Interestingly, the results demonstrated that IFN-*γ*, LPS, and Glu could significantly decrease the expression levels of CD73, while TNF-*α* did not lead to significant alterations of CD73 levels [63]. In addition, IFN-*α* and IFN-*β* have also been reported that they have an up-regulating effect on CD73 expression [64–66].

CD73 expression is also regulated by epigenetic modification. Lo Nigro et al. reported that CD73 expression was inhibited by its CpG island methylation in breast carcinoma cell lines and clinical tumor tissues [67]. Similarly, Wang et al. showed that CD73 expression was downregulated by methylation-dependent transcriptional silencing in melanoma cell lines. And in clinical cases of melanoma, methylation in the CD73 CpG islands was correlated with downregulated CD73 expression and was associated with metastatic site specificity [9].

Finally, some other studies have also reported that Wnt pathway [68], polyunsaturated fatty acid (PUFA) [69], and intracellular cAMP are also involved in regulation of CD73 expression [70]. Taken together, these results demonstrate that expression levels of CD73 are regulated by various factors via different mechanisms.

## 9. CD73 Targeting Therapy in Cancer

The emerging roles of CD73 in tumor growth and metastasis, especially as a key immunosuppressive factor in tumor microenvironment, have presented potential opportunities to develop anti-CD73 therapy for various human cancers. In this regard, accumulating results with small molecular inhibitors, or monoclonal antibodies targeting CD73 in mice tumor models, suggest that targeted CD73 therapy is an important approach to effective control of tumor growth and metastasis. Recently, Hausler et al. reported that anti-CD73 mAb enhanced the lytic activity of polyclonal NK cells against human ovarian cancer cell lines (SKOV-3 and OAW-42). Likewise, blocking of CD73 by mAb significantly increased proliferation of CD4^+^ T cells in coculture with ovarian cancer cells [71]. Thus, anti-CD73 treatment improves the immune cytotoxicity response against ovarian cancer cells. Stagg et al. found that anti-CD73 mAb therapy significantly delayed 4T1.2 and E0771 (two cell lines expressed high levels of CD73) primary tumor growth in immune-competent mice models, while it was ineffective in severe immunodeficient mice, which suggests that anti-CD73 mAb therapy requires adaptive immunity [11]. Obviously, above results demonstrated that anti-CD73 therapy was essentially dependent on its promotive effect on antitumor immune responses* in vivo*.

Cancer immunotherapy has becoming interesting in recent years. Drugs like anti-PD1 mAb and anti-CTLA-4 mAb which block programmed death-1 (PD-1) and cytotoxic T lymphocyte antigen (CTLA-4) receptors have shown very impressive objective response in patients [72]. Interestingly, Allard found that anti-CD73 mAb significantly enhanced the activity of both anti-CTLA-4 and anti-PD-1 mAbs against MC38-OVA (colon) tumor, RM-1 (prostate) subcutaneous tumors, and metastatic 4T1.2 breast cancer in mice models [73]. Recently, Lannone et al. reported that combination treatment with both APCP and anti-CTLA-4 mAb displayed significant retard of tumor growth compared with APCP or anti-CTLA-4 mAb treatment alone in mouse melanoma models [74].

Taken together, despite the anti-CD73 therapy has not been translated into clinical cancer patients, these results in tumor-bearing mice models have revealed that targeted blockade of CD73 may be an alternative and realistic therapeutic approach for cancer patients in the future. Human immune response is a complex process* in vivo*, besides CD73 mediated immunosuppression, tumor may also employ other mechanisms to facilitate immune escape. In consideration of this, combined use of drugs which target different immunosuppressive molecules may be useful therapeutic strategies for cancer patients in the future.

## 10. Conclusion

In summary, CD73 plays multiple roles in cancer related processes. The connection between CD73 overexpression and cancer subtype, prognosis, drug response of patients has presented the potential value of CD73 that served as a detectable biomarker in the coming age of individual cancer therapy. In addition, the promotive effect of CD73 on tumor growth and metastasis suggests that CD73 is a potential therapeutic target for cancer treatment. Targeting CD73 therapy with inhibitor or mAb has displayed favorable antitumor effects in mice tumor models. Combination treatment with both CD73 blockade and other developed immune-therapeutic agents (anti-CTLA-4 mAb, anti-PD1 mAb) seems especially attractive. These observations present a good opportunity to develop anti-CD73 therapy for the treatment of certain cancer patients. Based on these knowledge, although there are still a long way to go, future studies aiming at translating anti-CD73 therapy into clinical cancer patients can be expected. Notably, because of the multiple functions of CD73* in vivo*, although adverse events has not yet been observed in anti-CD73 treated mice models, the potential toxic risk of anti-CD73 therapy must be argued carefully before translating this therapeutic approach into cancer patients.

## Figures and Tables

**Table 1:** CD73 expression in different cancer cell lines.

Cancer type	Cell line	Expression	Reference
Glioblastoma Multiforme	T98G	Higher level of mRNA and protein than normal glial cell line SVGp12	[8]

Melanoma	C8161, PMW, SBCL2, SKMel28, SKMel30, KMel147, SKMel173, Mel224, WM266-4, M902-6	Higher mRNA expression than WM35 et al. because the absent of methylation	[9]

Melanoma	WM35, Mel501, Mel505, SKMel2, SKMel23, C81-61	Lower mRNA expression caused by the NT5E CpG island methylation	[9]

Melanoma	A375	High expression levels and activity	[10]

Mouse breast cancer	4T1.2, E0771	Higher expression levels than nonmetastatic variant of 4T1.2	[11]

Ovarian cancer	SKOV3	SKOV3 human ovarian cancer cells highly expressed CD73	[12]

Breast cancer	MB-MDA-231, T-47D	T-47D with lower expression of CD73 and MB-MDA-231 with higher expression of CD73	[13]

Primary Medulloblastoma Metastatic Medulloblastoma	Daoy, ONS76 D283	Daoy and ONS76 express higher levels of CD73 while D283 revealed poor expression of CD73	[14]

Bladder cancer	RT4, T24	Both cell lines expressed CD73	[15]

**Table 2:** The clinical significance of CD73 in cancer patients.

Cancer type	Number of patients	Clinical implication of CD73	Reference
Colorectal cancer	358	Overexpression of CD73 is an independent poor prognostic biomarker for human CRC	[16, 17]

Epithelial ovarian carcinoma	167	Overexpression of CD73 in epithelial ovarian carcinoma is associated with better prognosis, lower stage, and better differentiation	[18]

Gastric cancer	68	CD73 overexpression was positively correlated with differentiation of tumor, histopathology, depth of invasion, nodal status, metastasis, cancer stage, and low overall survival rate of patients	[19]

Gallbladder cancer	108	Survival time of patients with NT5E expression was significantly shorter than those without NT5E expression	[20]

Leukemia	86	Expression of CD73 was associated with leukemia subtype, differentiation, and development	[21]

Chronic lymphoblastic leukemia	229	High expression of CD73 was associated with a more aggressive and proliferation disease	[22]

Acute lymphoblastic leukemia (children)	338	CD73 expression had no prognostic value in children with acute lymphoblastic leukemia	[23]

Prostate cancer	116	Overexpression of CD73 in prostate cancer is associated with lymph node metastasis	[24]

Malignant melanoma	52	CD73 expression is epigenetically regulated in malignant melanoma and associated with metastatic site specificity	[9]

Breast cancer (stages I–III)	136	Elevated CD73 expression in stages I–III breast cancer can predict a good prognosis	[25]

Triple negative breast cancer Luminal breast cancer HER2+ breast cancer	661 2083 487	CD73 gene expression was significantly associated with a worse prognosis in TNBC patients but not in patients with luminal or HER2+ breast cancer	[26]
